# Women’s Cardiovascular Disease and Stroke Risk Stratification Using a Precision and Personalized Framework Embedded with an Explainable Artificial Intelligence Paradigm: A Narrative Review

**DOI:** 10.3390/diagnostics16081158

**Published:** 2026-04-14

**Authors:** Ekta Tiwari, Dipti Shrimankar, Mahesh Maindarkar, Luca Saba, Jasjit S. Suri

**Affiliations:** 1 Department of Computer Science and Engineering, Vishvswarya National Institute of Technology, Nagpur 440010, India; ekta.tiwari03@gmail.com (E.T.); dshrimankar@cse.vnit.ac.in (D.S.); 2 School of Bioengineering and Sciences & Research, MIT Art Design and Technology University, Pune 4123018, India; mahesh.nehu.333@gmail.com; 3 Department of Pathology, Azienda Ospedaliero Universitaria, 09124 Cagliari, Italy; 4 Department of Innovation, Global Biomedical Technologies, Inc., Roseville, CA 95661, USA; 5 Department of Electrical and Computer Engineering, Idaho State University, Pocatello, ID 83209, USA; 6 Symbiosis Institute of Technology, Nagpur Campus, Symbiosis International (Deemed University), Pune 440008, India; 7 Stroke Diagnostic and Monitoring Division, AtheroPoint™, Roseville, CA 95661, USA

**Keywords:** women’s CVD risk stratification, hormonal factors, pregnancy complications, autoimmune diseases, carotid ultrasound, machine learning and deep learning

## Abstract

**Background**: Women face underdiagnosed cardiovascular disease (CVD)/stroke risks due to sex-specific pathophysiological mechanisms, including hormonal variations such as oestrogen decline, adverse pregnancy outcomes (APOs), endothelial dysfunction, autoimmune-mediated factors, and sexual dimorphism in cardiac remodelling. Conventional risk assessment tools, predominantly calibrated to male pathophysiology, lack sensitivity in detecting these female-specific determinants. We hypothesise that artificial intelligence (AI), machine learning (ML) and deep learning (DL) may offer a transformative approach by integrating multimodal data, including pathological biomarkers, clinical history, and vascular imaging, to enable precision CVD/stroke risk stratification, pending rigorous external validation in sex-stratified cohorts. **Method**: This narrative review adopts a PRISMA-informed study selection framework and oversees gender-specific biomarkers, including vasoactive peptides (adrenomedullin), adipocytokines (adiponectin), inflammatory mediators (hs-CRP, IL-6), and thrombogenic factors (homocysteine, D-dimer), alongside clinical variables (APOs, autoimmune disorders) and ultrasonographic markers, carotid intima-media thickness (cIMT), plaque burden and plaque area (PA). Advanced ML/DL algorithms were employed to synthesise these heterogeneous datasets, identifying nonlinear interactions for better outcomes. **Findings**: Key insights reveal that hormonal dynamics (e.g., hypoestrogenism post-menopause) modulate CVD risk, while APOs induce persistent endothelial dysfunction and subclinical atherosclerosis. Biomarker sexual dimorphism is evident; hs-CRP exhibits higher baseline levels in women, whereas adiponectin declines with metabolic dysfunction. Radiomic features (cIMT progression, plaque morphology) are a well-established biomarker for CVD risk stratification. **Conclusions**: The integration of AI-driven multimodal systems holds the potential to enable a paradigm shift from population-based to personalised risk assessment, addressing critical gaps in female CVD health. However, this potential is currently at the early validation stage, and widespread clinical implementation requires prospective, externally validated, and ethnically diverse studies. Future applications should incorporate longitudinal biomarker profiling and advanced imaging, namely shear wave elastography and plaque radiomics, to optimise predictive models.

## 1. Introduction

Cardiovascular diseases (CVD)/Stroke are the most significant causes of mortality among women globally, yet prevention and treatment strategies have historically been derived from research focused on men [1]. Consequently, sex specific physiological and hormonal factors influencing women’s CVD/Stroke health are often overlooked [2,3]. These factors include a smaller heart size, narrower coronary arteries, and hormonal fluctuations, which contribute to distinct pathways in the development of CVD/Stroke [4]. This emphasises the necessity of specialised preventive and treatment strategies for women’s CVD/Stroke health [5]. Numerous studies have reported a *J-shaped* relationship between parity and CVD/Stroke risk, with women who have had two births exhibiting the lowest risk [6,7,8]. In contrast, nulliparous women and those with high parity (more than five births) demonstrate an elevated risk of heart disease, independent of breastfeeding history [9]. Furthermore, parity may serve as a surrogate marker for underlying socioeconomic, behavioural, and lifestyle factors that indirectly influence long-term cardiovascular health [4,10,11]. Pregnancy induces lasting physiological changes, such as weight gain and vascular stress, which may increase long-term CVD risk [12,13]. Further studies are required to elucidate these associations [14]. The global market for Artificial Intelligence (AI) in healthcare is projected to grow significantly, with AI-driven CVD and stroke solutions anticipated to account for 15–20% of this growth by 2030 [15]. A growing niche in women-specific CVD prevention is expected to capture 5–10% of this market, driven by demand for precision and personalised medicine [16,17,18].

AI has demonstrated methodological promise for personalised and accurate risk assessments, addressing novel risk factors such as hormonal changes [19]. However, most AI-based CVD risk stratification studies to date have been conducted in mixed-sex or predominantly male cohorts, and their demonstrated predictive benefit in women-specific populations remains limited. Claims of clinical improvement in this review should be interpreted in this context.

**Figure 1:**
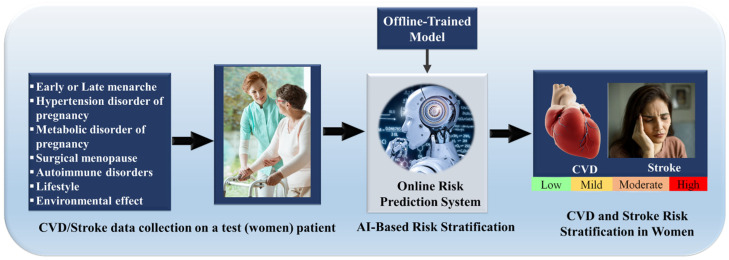
Symptoms in women (block 1) that lead to CVD/Stroke, and AI-based intervention guided by an offline trained model (block 2), leading to CVD risk stratification (block 3). (Some graphical elements (human illustrations) were obtained from Shutterstock (www.shutterstock.com) under a commercial license).

Oestrogen protects cardiovascular health during reproductive years by supporting vascular function and favourable lipid profiles [20,21,22,23]. Its decline post-menopause markedly increases the risk of CVD/Stroke [24,25]. Hormone replacement therapies (HRTs), while potentially helpful, have varied effects on cardiovascular health depending on individual health profiles, adding another layer of complexity [26]. Figure 1 shows the symptoms in women that lead to CVD/Stroke and ways in which AI-based intervention can be of use. Significant cardiovascular risks are also associated with pregnancy-related disorders such as gestational diabetes, pre-eclampsia, and pregnancy-induced hypertension [27,28]. These complications are major predictors of future CVD/Stroke, but are often underrepresented in traditional risk models [29,30].

Identifying these conditions early offers a critical opportunity for intervention to reduce long-term cardiovascular morbidity [31,32]. Autoimmune diseases, which are more common in women, further elevate CVD/Stroke risk. Chronic inflammation from conditions like systemic lupus erythematosus (SLE) and rheumatoid arthritis (RA) accelerates vascular damage and increases the likelihood of atherosclerosis [27,33]. Unfortunately, these inflammatory conditions are rarely included in traditional risk prediction models, leaving gaps in the care for CVD/Stroke risk in women [29,30]. The interactions among hormonal changes, inflammatory markers, and vascular health often follow non-linear patterns that conventional models struggle to capture [31,32,34].

Traditional CVD/stroke risk calculators, such as the Framingham Risk Score (FRS), Systematic Coronary Evaluation Score (SCORE), and Atherosclerotic Cardiovascular Disease (ASCVD) risk estimator, frequently fail to accurately predict CVD/stroke risk in women using either external or internal risk factors [35,36]. This is because they make linear assumptions about risk factors and outcomes [37,38]. This limitation can result in underestimating risk under complex conditions like autoimmune disorders, OSA, Parkinson’s disease [39,40,41], obesity [42], foot disease [43,44], erectile dysfunction [26], and extensive plaque burden [45,46]. Advanced AI models that fuse clinical biomarkers, laboratory data, and imaging techniques can better handle ‘J-shaped’ nonlinear interactions, providing more accurate predictions [42,46,47,48]. These sophisticated tools have transformative potential in cardiology, radiology, and related fields by extracting intricate features from medical data [41,43,49,50].

This narrative review is organised around three primary thematic pillars: (i) female-specific hormonal and physiological determinants of CVD/stroke risk; (ii) pregnancy-related conditions and autoimmune diseases as amplifiers of sex-specific cardiovascular risk [51]; and (iii) AI and ML methodologies for integrating these heterogeneous risk factors into personalised risk stratification frameworks [11]. Autoimmune diseases are examined as an important modifier of women’s CVD risk rather than as the central focus of the review.

## 2. PRISMA-Informed Literature Selection Strategy

CVD/Stroke is the leading cause of morbidity and mortality among women, characterised by distinctive risk profiles shaped by hormonal, lifestyle, and socio-environmental factors in addition to structural changes. This narrative review consolidates current evidence on these multifaceted risks, highlighting the interplay between internal, external, and combined factors influencing women’s cardiovascular health.

**Figure 2:**
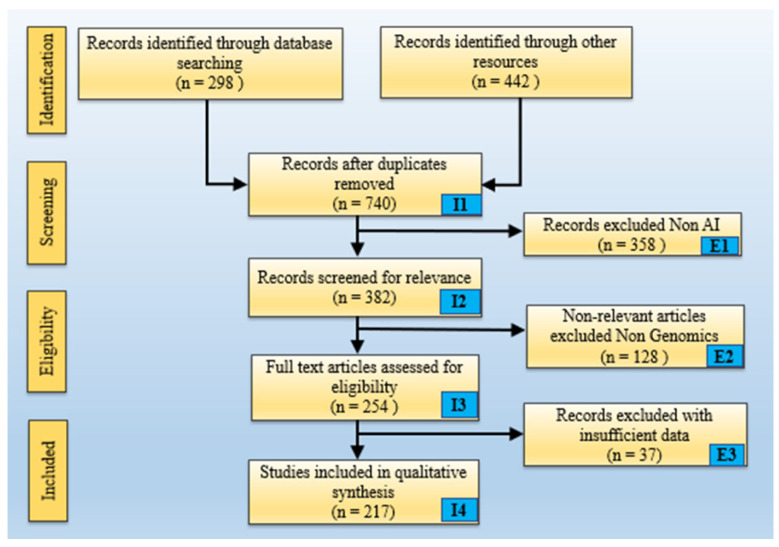
PRISMA model for literature selection strategy [I: Included, E: Excluded].

This narrative review adopts a PRISMA-informed framework to ensure a transparent and reproducible approach to literature identification, screening, and synthesis; however, it does not constitute a formal systematic review or meta-analysis. No protocol was prospectively registered, no formal risk-of-bias appraisal was conducted, and inter-rater reliability for study selection was not assessed. The PRISMA flow diagram (Figure 2) reflects the process by which studies were identified and selected for narrative synthesis, and should be interpreted accordingly. The PRISMA methodology involves four main stages: (i) identification, (ii) screening, (iii) eligibility, and (iv) inclusion, detailed as follows:

*i*. *Identification*: A comprehensive search was conducted using electronic databases, including Google Scholar, Web of Science, PubMed, and Scopus. Peer-reviewed journal articles, conference proceedings, and review papers concentrating on risk factors for CVD/Stroke in women were included.
*ii*. Keywords and Medical Subject Heading (MeSH) terms included combinations of “women’s cardiovascular health,” “hormonal influences,” “autoimmune diseases and pregnancy-related complications,” “CVD and stroke,” “socioeconomic determinants,” and “artificial intelligence in risk stratification”, “Standard LSTM”, “Bidirectional LSTM (BiLSTM)”, “Stacked LSTM (SS-LSTM)”, “Gated Recurrent Unit (GRU)”, Boolean operators (AND, OR) and truncation were employed to refine the search strategy. Citation tracking and reference list scanning of applicable articles were performed to identify additional studies.
*iii*. *Screening*: The reclaimed articles were screened based on titles and abstracts. Replicated records were removed. Studies were included if they (a) focused on risk factors specific to women’s cardiovascular health or stroke. (b) Addressed the use of advanced diagnostic or predictive tools, such as AI-driven models. (c) Provided data from observational, longitudinal, or interventional studies. (d) Non-English articles, case reports, editorials, and studies with insufficient data were excluded.
*iv*. *Eligibility*: The full texts of articles identified as potentially relevant were evaluated based on established inclusion and exclusion criteria. Articles were excluded if they did not specifically address gender differences in CVD/Stroke risk factors, lacked clear outcomes, or involved unrelated methodologies.
*v*. *Inclusion*: A total of 171 studies were included in the final review. These studies were synthesised to address the interplay of internal, external, and combined risk factors influencing CVD/Stroke in women. A PRISMA flow diagram summarising the selection process is presented in Figure 2.

## 3. Biological Link Between Biomarkers and CVD/Stroke Risk in Women

**Figure 3:**
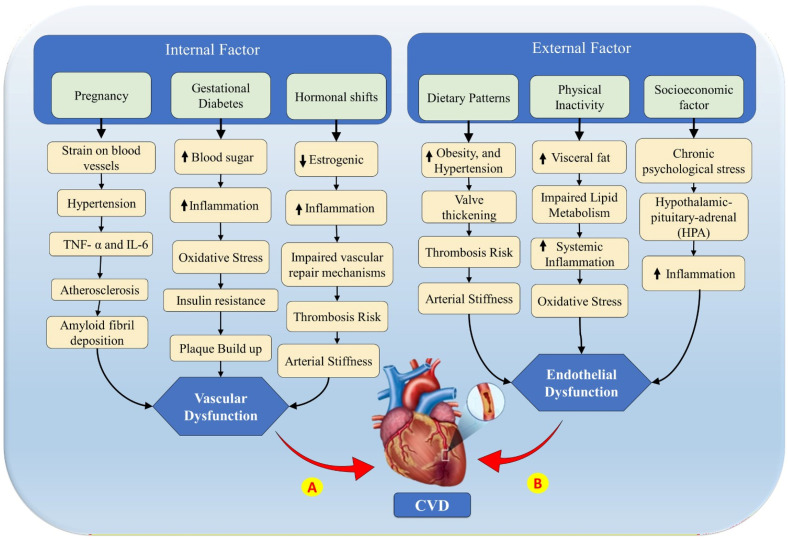
Internal and external risk factors in women patients leading to CVD. A: Pathway from vascular dysfunction (internal factors). B: Pathway from endothelial dysfunction (external factors). (Some graphical elements were obtained from Shutterstock (www.shutterstock.com) under a commercial license).

CVD/Stroke in women is regulated by complex interactions among biological, hormonal, lifestyle, and immune factors. These interconnected pathways, as depicted in Figure 3, highlight the dynamic interplay between external and internal risk factors that contribute to endothelial dysfunction, vascular dysfunction, and ultimately, cardiovascular disease risk outcomes. Addressing these factors is critical for developing precise prevention and management strategies tailored to women.

### 3.1. Hormonal Influences

Women’s cardiovascular health is heavily influenced by hormonal changes, particularly those involving oestrogen [52], as is supported by validated predictive evidence. During the reproductive years, oestrogen has been shown in longitudinal studies to protect against CVD/Stroke by improving endothelial function, increasing vasodilation, and lowering inflammation [53,54]. It improves lipid profiles by increasing HDL cholesterol while decreasing LDL cholesterol, thereby reducing the risk of atherosclerosis [55,56], as is substantiated by mechanistic and prospective epidemiological evidence. Following menopause, the dramatic fall in oestrogenic levels has been prospectively associated with arterial stiffness, increased vascular inflammation, and reduced vascular repair processes, associations that appear to translate into significantly increased risk of CVD [57,58]. Corroborated by observational/associative evidence, a mechanistic basis has been established. Hormone replacement therapy (HRT) may have varying cardiovascular effects depending on individual health profiles and treatment timing [59,60], as reinforced by associative evidence from randomised and observational studies, although this has not been uniformly validated across populations. Pregnancy-related hormonal alterations, including those associated with hypertension, pre-eclampsia, and gestational diabetes, can affect vascular and metabolic processes and serve as early indicators of future CVD/Stroke risk [61,62].

### 3.2. Autoimmune Diseases as Amplifiers of CVD Risk in Women

Autoimmune diseases such as systemic lupus erythematosus (SLE) and rheumatoid arthritis (RA), confirmed by associative evidence from prospective cohort studies, which primarily affect women, are cross-sectionally associated with chronic inflammation, which is a recognised contributor to endothelial dysfunction and vascular remodelling [63]. While these associations are well-documented, their independent predictive value in CVD risk models beyond traditional risk factors has not been fully established and warrants further longitudinal investigation [64]. It has also been confirmed by mechanistic evidence that autoantibodies such as anti-citrullinated peptide antibodies (ACPAs) and pro-inflammatory cytokines, including TNF-α and IL-6, could aggravate these disease processes [65,66]. Chronic inflammation associated with these disorders hastens arterial damage, increasing the risk of atherosclerosis and myocardial infarction in women [62,67]. Traditional risk models frequently overlook these elements, necessitating the use of advanced technologies for accurate risk prediction and management [68].

### 3.3. Physiological and Anatomical Differences

Women’s distinctive cardiovascular structure, which includes a smaller heart and thinner coronary arteries, influences disease presentation and outcomes [69]. These structural changes cause more diffuse atherosclerosis and unusual symptoms, frequently delaying diagnosis and treatment [70]. Smaller coronary arteries are more prone to blockage, emphasising the importance of accurate imaging techniques adapted to these anatomical differences [71].

### 3.4. Pregnancy as a Window to Cardiovascular Health

Pregnancy represents a critical period for unmasking latent cardiovascular vulnerabilities [72]. Complications such as pre-eclampsia, gestational diabetes, and pregnancy-induced hypertension induce systemic inflammation, oxidative stress, and hypercoagulability, all of which are precursors to long-term CVD/Stroke morbidity, as is corroborated by mechanistic and associative evidence; longitudinal predictive evidence is available for pre-eclampsia [73,74]. Women with a history of these conditions are at a significantly higher risk of developing hypertension and ischemic heart disease later in life, emphasising the importance of early monitoring and intervention, as is validated by predictive evidence and supported by prospective longitudinal cohort data [75,76].

### 3.5. Interplay and Non-Linearity of Risk Factors

The interplay among hormonal changes, chronic inflammation, and pregnancy-related complications demonstrates a non-linear relationship with cardiovascular outcomes [59,77]. For example, oestrogenic deficiency post-menopause amplifies inflammatory pathways, further elevating CVD/Stroke risk in women with autoimmune disease (Mechanistic evidence; clinical translation not yet formally validated in predictive models) [78,79]. Similarly, pregnancy complications may synergies with pre-existing metabolic disorders, compounding long-term risks substantiated by from observational studies [80,81]. Recognising these patterns is essential for developing effective, personalised and preventive strategies. Table 1 shows various biomarkers that lead to CVD/Stroke events in women’s patients.

## 4. Classification of Risk Factors for CVD/Stroke in Women

Figure 3 classifies CVD/Stroke risk factors into external, internal, and combined categories, illustrating the pathways leading to vascular dysfunction (Pathway A) and endothelial dysfunction (Pathway B).

### 4.1. Internal Factors

Internal factors are intrinsic to the individual and include biological, hormonal, and genetic variables [82]. Hormonal variations, particularly involving oestrogenic, protect against vascular inflammation and maintain endothelial integrity during reproductive years, validated by mechanistic and observational evidence [83].

However, the decline in oestrogen post-menopause increases the risk of CVD/Stroke, as is corroborated by associative evidence; thus, a biologically plausible mechanism has been established [84,85]. Pregnancy-related conditions such as pre-eclampsia, gestational diabetes, and pregnancy-induced hypertension significantly disrupt vascular and metabolic processes, serving as early markers for future CVD/Stroke [86,87]. Autoimmune diseases further amplify risk through chronic inflammation and accelerated vascular damage [88,89]. Biomarkers such as carotid intima-media thickness (cIMT), plaque burden/area (PA), rheumatoid factor (RF), and ACPAs provide measurable insights into disease progression [90,91]. It should be noted that while RF and ACPAs are validated markers of autoimmune disease activity, their independent contribution as predictors within CVD/stroke risk models remains an area of active investigation, and their inclusion in traditional risk scores is not currently standard practice [92,93].

Several biomarkers shown in Table 2, including CRP/hs-CRP, IL-6, and D-dimer, represent systemic inflammation and thrombotic risk and play important roles in the development of CVD, T2DM, and cancer, which, for most of these, is reinforced by associative evidence; hs-CRP has limited validated predictive evidence in women-specific CVD risk models [94]. Dyslipidaemia indicators, such as LDL, HDL, and total cholesterol, improve stratification by identifying common cardiometabolic risk profiles (validated predictive evidence in traditional and AI-augmented risk models) [95,96].

### 4.2. External Factors

External factors encompass lifestyle, environmental, and social determinants of health. Sedentary behaviour, unhealthy dietary patterns, tobacco use, and psychosocial stressors are major contributors to CVD/Stroke risk in women [97,98]. Socioeconomic differences and poor healthcare access amplify these risks, especially among marginalised groups [99,100].

Environmental exposures, such as air pollution and occupational stress, exacerbate these consequences [101,102]. These variables interact with internal vulnerabilities, accelerating illness progression and resulting in negative effects.

### 4.3. AI-Driven Insights

**Figure 4:**
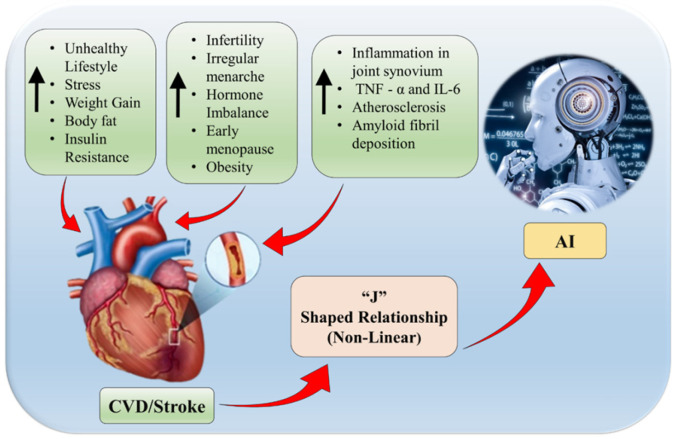
The nonlinear nature of various CVD/Stroke biomarkers in women patients and AI. (Some graphical elements were obtained from Shutterstock (www.shutterstock.com) under a commercial license).

AI models are preferably suited to analysing the connections between internal and external elements. These models can identify complex patterns by integrating data from several sources, allowing for more personalised preventative management efforts [103]. The pathways shown in Figure 4 demonstrate the diverse and allied nature of CVD/Stroke risk factors in women patients. Considering these interactions is critical for creating personalised interventions that address internal susceptibilities and changeable external factors [46,71,83]. These impacts are exacerbated by environmental exposures such as air pollution and work-related stress [101]. These elements compound illness progression and negative consequences by interacting with internal susceptibilities.

The biological and environmental interplay of risk factors in women underscores the need for a comprehensive and nuanced approach to CVD/Stroke prevention and management [63,104]. By categorising these risks into internal, external, and combined factors and addressing their non-linear interactions, healthcare providers can better target interventions. AI may offer methodological advantages in integrating and analysing these multifaceted risks—potentially improving personalisation and precision in CVD/Stroke risk assessment for women, although this is subject to validation in sex-stratified cohorts [41]. This paradigm shift represents a critical step toward addressing the unique challenges of women’s CVD/Stroke complications.

## 5. Artificial Intelligence’s Significance in Women’s CVD/Stroke Risk Identification

ML and DL techniques have been applied to CVD/stroke risk prediction across a range of clinical contexts [41,50,105,106,107,108,109,110]. For instance, Jamthikar et al. [90] employed an ML-based system integrating carotid ultrasound phenotypes and conventional risk factors in an Asian–Indian cohort (*n* = 200), reporting improved risk stratification versus Framingham Risk Score alone—though this study did not include a women-only subgroup analysis. Konstantonis et al. [32] demonstrated the utility of ML with carotid/femoral imaging in rheumatoid arthritis patients, a population that was disproportionately female, but external validation in an independent cohort was not reported. It is important to note that most published DL-based CVD risk models were developed based on mixed-sex datasets, and specific performance metrics in women-only cohorts remain sparse in the literature. Where validation data are available, they are referenced explicitly; where they are absent, findings should be interpreted as proof-of-concept rather than established clinical evidence.

A strong framework for risk stratification is produced by combining ML and DL techniques [111,112,113]. With an emphasis on appropriate dataset preparation and organisation for training and testing, our team has thoroughly investigated DL applications [32,114]. Four essential processes are involved in this approach: (i) pre-processing the data for quality control; (ii) dividing the data into augmented training and non-augmented testing data; (iii) creating training models while maintaining generalisation; and (iv) using the test data to predict the risk of CVD/stroke [105,115,116]. Adaptive Synthetic Sampling (ADASYN) [109], Synthetic Minority Over-Sampling Technique (SMOTE) [32,107], and principal component analysis (PCA)-based pooling for feature selection [115,117,118] are examples of normalisation and augmentation approaches used in preprocessing. To provide a trustworthy evaluation, K10 cross-validation separates data into training and testing sets.

**Figure 5:**
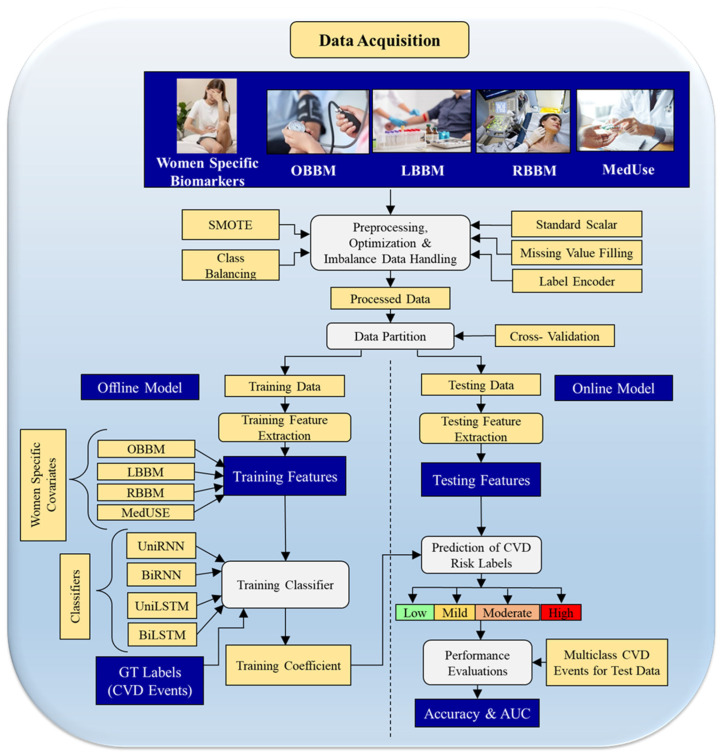
ML/DL system for women patients’ CVD and stroke risk stratification [Genomics-based, proteomics-based, office-based, laboratory-based, radiomics-based, and GBBM-based biomarkers, MedUse: Pharmaceuticals, CVD: Cardiovascular illness, BiLSTM: Bidirectional Long-Short Term Memory, UniLSTM: Unidirectional Long-Short Term Memory, BiRNN: Bidirectional Recurrent Neural Networks, UniRNN: Unidirectional Recurrent Neural Networks]. (Some graphical elements (human illustrations) were obtained from Shutterstock (www.shutterstock.com) under a commercial license).

DL classifiers that take advantage of different risk factors, such as recurrent neural networks (RNNs) and long short-term memory (LSTM) models, are used to build offline coefficients [25,39,109]. Predictions are refined by avoiding data overlap between training and testing sets, ensuring accuracy, reproducibility, and generalisation [105,115,116]. Embedded feature optimisation enhances learning, and live systems evaluate performance metrics like accuracy, sensitivity, specificity, recall, area under the curve (AUC), and *p*-values [45]. To estimate CVD/stroke risks in women with autoimmune diseases, this thorough approach combines ML and DL algorithms with stringent preprocessing, accurate segmentation, and performance evaluation (Figure 5). Biomarkers such as office-based biomarkers (OBBM), laboratory-based biomarkers (LBBM), radiomics-based biomarkers (RBBM), genomics-based biomarkers (GBBM), medication usage (MedUSE), and autoimmune symptoms are included in the input data [119]. This integrated methodology aligns ML and DL approaches with rigorous preprocessing, accurate segmentation, and performance evaluation to estimate CVD/Stroke risk in women with autoimmune diseases (Figure 5). OBBM, LBBM, RBBM, GBBM, MedUSE, and autoimmune symptoms are among the biomarkers included in the input data [120].

### 5.1. CVD/Stroke Risk Stratification Using ML-Based Classifiers

ML classifiers are instrumental in categorising patients into low- or high-risk groups for CVD/stroke. In the populations studied, which were predominantly mixed-sex, Random Forest (RF)-based approaches demonstrated improved stratification over conventional risk scores; replication in women-specific cohorts with external validation is required before this advantage can be generalised [121,122]. In the context of cardiovascular risk in women, RF-based models require evaluation in sex-stratified datasets with appropriate calibration reporting before their advantages can be generalised beyond the populations in which they were originally developed [123]. Recent studies also highlight surrogate biomarkers and DL solutions in RA for CVD/Stroke risk prediction [124,125].

### 5.2. CVD/Stroke Risk Stratification Using Uni-Bidirectional RNN and LSTM-Based Classifiers

Recurrent Neural Networks (RNNs) and Long Short-Term Memory (LSTM) networks have shown promise in tabular data for medicine tasks, hypothesising that subtle changes between patients act like a temporal change in disease growth. Thus, the spatial patient representation acts like a temporal sequence under the RNN framework. Such a paradigm is powerful for CVD risk stratification [126,127]. In unidirectional architectures, these models process information in a single direction, typically from earlier to later frames, thus leveraging only past context during prediction [128]. Direct application of these architectures to women-specific CVD risk cohorts remains limited in the published literature; most validation studies have been conducted in general or mixed populations. Future work should specifically examine the performance of these models in longitudinal women’s health datasets, where hormonal trajectories and pregnancy history provide unique time-series patterns.

However, bidirectional RNNs and LSTMs enhance performance by processing image sequences in both forward and backward directions, effectively capturing contextual information from the entire sequence [129]. While this architecture has demonstrated performance advantages in general clinical settings, its specific application to women’s CVD risk datasets remains limited, and published results should be interpreted within the constraints of the study populations from which they were derived [130].

**Figure 6:**
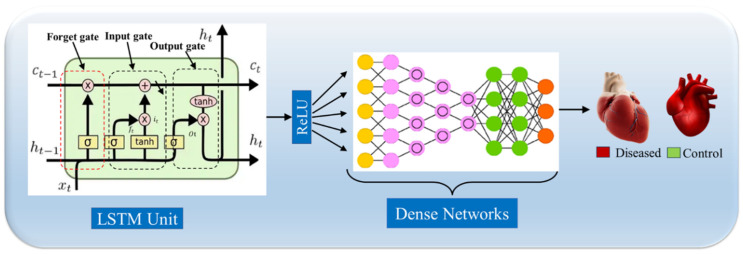
The fundamental design of an LSTM structure with dense networks [43]. (Some graphical elements were obtained from Shutterstock (www.shutterstock.com) under a commercial license).

LSTMs handle long-term dependencies, identifying temporal patterns that are critical for risk estimation (Figure 6). Despite their effectiveness in time-series analysis, the efficacy of LSTM networks remains limited, particularly for long-term electrocardiogram (ECG) event prediction [131]. To address these issues, the next section provides a brief overview of upgraded LSTM designs that aim to improve long-range dependency modelling and prediction accuracy [132].

ECG represents a high-resolution cardiac electrical signal, whereas cardiovascular datasets encompass broader multimodal clinical and physiological information used for population-level risk assessment and disease prediction.

### 5.3. Enhanced LSTM Architectures for Biomedical Sequence Modelling for Risk Stratification

LSTM with Context-Fusion (cLSTM) extends the normal LSTM by introducing an external context vector, such as patient demographics, lab data, or comorbidities, directly into the gated computations [133]. This allows the model to concurrently learn temporal patterns and patient-specific variables, boosting personalisation and risk-stratified predictions [134]. Extended Long Short Term Memory with Cross-Gating (xLSTMcg) allows gates to communicate with one another instead of working individually, and this improves the sequential modelling. The architecture works well for noisy biomedical signals like ECG, Electroencephalogram (EEG), and Photoplethysmogram (PPG) because these cross-connections enhance selective information flow, lower noise, and stabilise long-range dependency [135]. Multi-Headed Gated Attention LSTM (xLSTMega) combines multi-head gated attention with an expanded LSTM controller.

Multiple attention heads record both local and global temporal patterns, and adaptive memory control refines the representation. This allows for very expressive modelling of complicated, multimodal biomedical sequences, including EHR and illness progression data [136]. cLSTM adds context awareness, xLSTMcg improves temporal selectivity through gate interactions, and xLSTMega provides enhanced multi-scale learning through gated attention, resulting in a gradual hierarchy of increased sequence-modelling capabilities for biomedical applications. The detailed figures are explained in Appendix A, the mathematical equations of LSTM families are shown in Appendix B, and a comparison of LSTM models is shown in Appendix C.

### 5.4. Generative Adversarial Networks

Generative Adversarial Networks (GANs) contribute to classification by augmenting datasets with synthetic samples, improving performance on imbalanced data [137]. These networks learn feature representations through generator–discriminator interplay, enhancing classification accuracy even with limited labelled data [138,139]. Applications include domain adaptation, privacy-preserving data fusion, and broader scenario modelling, offering robust solutions for CVD/Stroke risk prediction [140].

### 5.5. Model Pruning

The growing integration of edge technologies within mobile frameworks highlights the need for model compression to enable effective deployment in resource-constrained environments [141]. To address these challenges, optimisation techniques such as genetic algorithms (GA), differential evolution (DE), and particle swarm optimisation (PSO) have been employed to enhance the efficiency and accuracy of fully convolutional networks (FCN) and SegNet architectures [43]. These advancements make it feasible to deploy lightweight models, such as RBBM and GBBM, ensuring accessibility and scalability in rural and underserved regions where computational resources and connectivity may be limited [142].

## 6. Artificial Intelligence Explainability

In the proposed precision and personalised framework, the primary data modality for CVD/Stroke risk stratification in women is structured, tabular data derived from clinical, biochemical, hormonal, reproductive, autoimmune, and surrogate vascular biomarkers. These datasets typically include demographic variables, menopausal status, adverse pregnancy outcomes (APOs), inflammatory markers (e.g., hs-CRP, IL-6), metabolic indices, autoimmune markers, medication usage, and ultrasound-derived quantitative features such as carotid intima-media thickness (cIMT) and plaque burden.

While advanced ML and DL models trained on such heterogeneous data can capture complex non-linear relationships and interactions that are not adequately modelled by conventional statistical approaches, their lack of transparency presents a significant barrier to clinical translation. In this context, explainable artificial intelligence (XAI) methods such as Shapley Additive Explanations (SHAP) and Local Interpretable Model-Agnostic Explanations (LIME) are essential for elucidating the decision-making processes of these predictive models and aligning algorithmic outputs with clinical reasoning. From a theoretical perspective, LIME approximates the complex, non-linear decision boundary of a black-box model with a locally interpretable surrogate model in the vicinity of a specific prediction [143]. Given an instance of interest, LIME perturbs the input feature space to generate synthetic samples around that instance and assigns weights based on proximity, thereby fitting a sparse linear model that faithfully represents the model’s behaviour within that local region [144]. This locality assumption is particularly suitable for heterogeneous clinical tabular data, where global model behaviour may be highly non-linear, but clinically meaningful explanations are required at the individual patient level. In the context of women’s CVD/stroke risk stratification, LIME enables clinicians to understand how specific combinations of biomarkers, such as inflammatory markers, hormonal status, and pregnancy-related variables, contribute to an individual’s risk estimate, without requiring access to the predictive model’s internal structure [145]. As a model-agnostic technique, LIME can be applied consistently across ensemble ML models and DL-derived risk scores, thereby supporting interpretability, clinical trust, and real-time decision support in personalised cardiovascular care [146,147].

**Figure 7:**
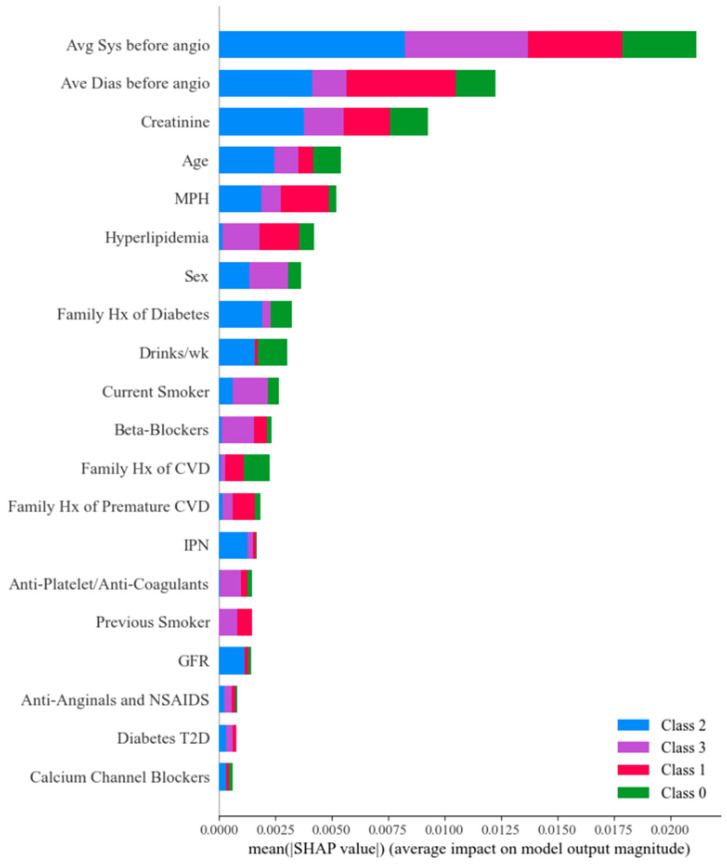
Class-wise SHAP values for xLSTMeg. xLSTMmg: extended Long Short-Term matrix gating; IPN: Intraplaque Neovascularization; TPA: Total Plaque Area; GFR: Glomerular Filtration Rate; BMI: Body Mass Index [130].

SHAP provides a principled, game-theoretic framework that decomposes individual CVD/stroke risk predictions into additive contributions from each input feature, thereby enabling both global and local interpretability [148,149]. Global SHAP analyses facilitate the identification of dominant women-specific risk drivers across populations, such as chronic inflammatory burden, hypoestrogenism, history of pregnancy complications, autoimmune disease activity, and dyslipidaemia, while local SHAPs enable patient-level attribution of risk scores, supporting personalised clinical decision-making. Importantly, SHAP interaction values allow explicit modelling of the synergistic and antagonistic effects between the features shown in Figure 7, reflecting the biologically non-linear interplay among hormonal changes, immune dysregulation, and vascular pathology that characterises female CVD/Stroke risk. LIME complements SHAP by constructing locally faithful surrogate models around individual predictions, offering intuitive and computationally efficient explanations that are particularly valuable in real-time clinical settings. Together, these XAI techniques may contribute to improved trust, bias detection, and transparency, which are considered necessary, though not sufficient, for regulatory acceptance and ethical deployment of AI-driven risk-stratification tools in women’s CVD/Stroke care. Their effectiveness in real-world clinical contexts requires empirical validation beyond the proof-of-concept settings in which they have primarily been studied.

### Limitations of Post Hoc XAI Methods

While SHAP and LIME provide valuable attribution information, several important limitations must be acknowledged. First, neither method establishes causal relationships between biomarkers and CVD/stroke outcomes; they describe the model’s learned associations, not the underlying biological mechanisms. Second, SHAP values are sensitive to feature correlation: in clinical datasets where biomarkers co-vary (e.g., inflammatory markers, lipid panels), SHAP attributions may distribute importance across correlated features in ways that are difficult to interpret clinically. Third, LIME’s local linear approximations are sensitive to the choice of neighbourhood radius and perturbation distribution, and may produce inconsistent explanations across similar patient instances. Fourth, in high-dimensional, data-limited settings that are characteristic of women-specific CVD research, both methods are subject to instability that can undermine confidence in individual-level explanations. XAI outputs should therefore be interpreted by clinical domain experts and viewed as decision support tools rather than authoritative causal explanations.

## 7. Future of CVD Risk Assessment in Women

**Figure 8:**
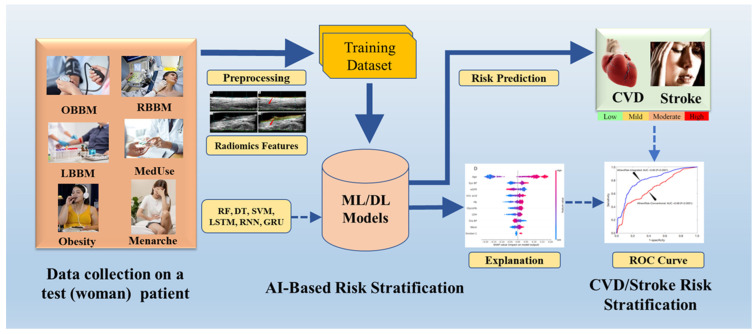
An ML/DL integrated system with biomarkers specific to CVD/stroke in women is incorporated into CVD/stroke risk stratification. LSTM: Long Short Term Memory, RNN: Recurrent Neural Network, GRU: Gated Recurrent Network, RF: Random Forest, DT: Decision Tree, SVM: Support Vector Machine) [124]. (Some graphical elements (human illustrations) were obtained from Shutterstock (www.shutterstock.com) under a commercial license).

In the area of women’s health, many factors cause CVD, including genetic, hormonal, reproductive, metabolic, and physiological factors [150,151,152]. The stratification of CVD risk is necessary in order to prevent disease. There are many existing methods that use ML and DL. But they are not yet reliable and not scientifically validated [153,154,155]. For this reason, there is a need for an advanced AI system that can predict risk scores, which is also scientifically validated. Figure 8 shows how CVD/Stroke risk stratification can be accomplished through the incorporation of an ML/DL-integrated system with women’s CVD/Stroke biomarkers.

The latest developments in Generative Artificial Intelligence (GenAI) and Transformer models (especially in risk stratification) also seem to address these challenges [156,157,158,159]. Using attention mechanisms, Transformers can be trained on and learn from an infinite number of nonlinear, complex relationships among various biomarkers [160,161,162]. These may include clinically, lab-, or imaging-derived radiomic metrics, and woman-specific hormonal variables, among others. Additionally, GenAI solutions can incorporate schema augmentation, learn latent representations, and develop uncertainty models, all of which are crucial in enhancing robustness in small and/or imbalanced female cohorts [163,164,165].

When developed in conjunction with biologically meaningful constraints and rigorous validation methodology, these AI tools could potentially produce more trustworthy CVD/Stroke risk scores for women—provided that external validation in sex-stratified cohorts is achieved and that calibration and fairness metrics are systematically reported. Transformers and GenAI models present an improvement on what we see in present machine learning and deep learning for women’s health in CVD/Stroke risk prediction [166,167]. Traditional models use fixed feature interactions and have a limited set of biomarkers, but Transformer-based architectures are able to identify long-range and cross-domain relationships in very different types of input data, from clinical info to lab results, imaging data, genetic info, and women-specific hormonal and reproductive data [168,169,170]. This provides a better picture of the very complex and nonlinear issues that play out in CVD/Stroke in women, which also include the diffuse and microvascular disease patterns that often are left out by conventional models. Also, GenAI improves performance by way of robust feature learning, creating synthetic data for underrepresented female groups, and in risk estimates that take uncertainty into account [171,172,173]. When paired with biologically informed constraints, existing ML/DL models help in improving generalisation, decreasing false positives in risk stratification, and yielding more consistent, interpretable, and stable risk scores [174,175].

These advances represent promising avenues for future investigation to improve on established clinical tools, and their translation will require rigorous, prospective, sex-stratified validation. Future research should prioritise the integration of multimodal datasets, the incorporation of women-specific biological insights, systematic external validation across diverse cohorts, and the development of clinically interpretable, scientifically validated tools to improve the translational reliability of CVD/Stroke risk assessment in women [176].

## 8. Critical Discussion

CVD/Stroke is the primary contributor to morbidity and mortality among women worldwide. Women face distinct risk factors, such as hormonal variation, pregnancy-related health issues, and the effects of chronic inflammatory disorders, which complicate the prediction and management of these conditions. Addressing these challenges, along with the interplay of non-linear risk factors, calls for sophisticated AI-based systems to enhance CVD/Stroke risk stratification and improve outcomes [177]. However, implementing such systems poses significant challenges, including data integration, accuracy, and clinical applicability.

### 8.1. Principal Findings

Three significant developments in women’s CVD/stroke risk categorisation are highlighted in this study:

*i*. Amalgamation of Diverse Biomarkers:

To quantify the risk of CVD/Stroke, office-based biomarker data, clinical biomarkers such as pathological biomarkers, and radiomics-based biomarkers derived from radiology images are all fused. This fusion technique highlights the importance of recording sex-specific changes to increase forecast accuracy. It should be noted that this integration approach has primarily been demonstrated in proof-of-concept studies; large-scale, externally validated applications in women-only cohorts remain to be developed [178,179].

*ii*. AI-Focused Predictive Modelling

A DL-based framework based on precision, predictive, and preventative medicine is a powerful paradigm for CVD/Stroke risk stratification among women; this method overcomes the inadequacies of conventional risk prediction methods.

*iii*. Carotid Imaging and Ample Data

This novel approach to risk assessment combines imaging and clinical biomarkers with carotid ultrasound imaging (cBUS) [40]. This all-encompassing method offers a strong framework for identifying early vascular alterations and classifying women’s risk for CVD/Stroke. Following the above developments, the study demonstrates the ability of AI-based systems to more efficiently diagnose CVD/Stroke risks and deliver targeted prevention methods.

### 8.2. Benchmarking Analysis

Vital studies on CVD/Stroke and the role of AI in women’s health, highlighting gender-specific factors as well as biological and lifestyle determinants and AI applications, are shown in Table 3. Readers should note that direct cross-study comparisons are limited by heterogeneity in study populations, feature sets, validation strategies, and outcome definitions. Several benchmarked studies were conducted in mixed-sex or predominantly male cohorts, and their findings may not generalise to women-specific settings. This analysis should therefore be interpreted as a descriptive landscape of current research activity rather than a formal comparative assessment of model performance.

Morales Lara et al. [180] explore women’s CVD/Stroke using AI and digital tools, proposing a broad perspective. In contrast, Suri et al. [39] apply DL to predict heart disease and stroke risks in Parkinson’s patients, focusing on a neurological comorbidity. This contrast underscores AI’s versatility across varied populations. Jonas et al. [181] examine coronary stenosis and plaque characteristics across age groups, while Ahmed, A. et al. [103] investigate sex-specific differences in ischemic total perfusion defect (ITPD), incorporating lifestyle and autoimmune factors.

Tamarappoo et al. [182] demonstrate the value of integrating diverse parameters for a deeper understanding of cardiovascular risks. Sun et al. [183] advocate for AI as a complete tool for CVD health management, whereas Jamthikar, A.D. et al. [107] focus on its clinical application in diagnostics. This comparison reflects dual AI pathways: broad population-level assessments and targeted clinical interventions. Building on this narrative, Yan et al. [184] provide a thorough assessment of AI’s contribution to bettering the diagnosis and treatment of CVD/stroke, comparing it to research by Webb et al. [185] and Abouzeid et al. [186]. These comparative studies examine risk variables and gender-specific issues influencing the health of women with CVD/Stroke.

**Table 3:** Benchmarking table. Ref: References, CVD: Cardiovascular disease, AI: Artificial Intelligence, Obs: Obesity, AD: Autoimmune disease. (✓: Included, ✗: Not included).

Ref.	Objectives	Obs	Pregnancy	Lifestyle	AD	CVD	Menarche	AI	AI Bias
Morales-Lara et al. [180]	Explored holistic frameworks integrating AI with digital health tools for gender-specific CVD insights.	✓	✓	✗	✗	✓	✓	✓	✗
Suri et al. [39]	Highlighted AI’s ability to extend to neurological comorbidities, bridging CVD/Stroke and neurological risks.	✓	✓	✓	✗	✓	✓	✓	✓
Jonas et al. [181]	Showcased AI’s capability to assess age-related structural changes in arteries, aiding early detection.	✓	✓	✗	✗	✓	✗	✓	✗
Tamarappoo et al. [182]	Integrated lifestyle and autoimmune factors, enriching models for sex-specific risk evaluation.	✓	✓	✓	✓	✓	✓	✓	✗
Sun et al. [183]	Demonstrated AI’s role in supporting cardiologists through advanced diagnostic analytics.	✓	✓	✓	✗	✓	✗	✓	✗
Hackman et al. [187]	Highlighted AI’s utility in clinical settings, focusing on precision and efficiency in diagnostics.	✓	✗	✗	✓	✓	✗	✓	✗
Yan et al. [184]	Presented systemic advancements in personalised care through AI-driven diagnostic enhancements.	✓	✓	✓	✗	✓	✓	✓	✗
Webb et al. [185]	Emphasised gender-specific barriers, supporting the need for tailored AI interventions.	✓	✓	✗	✓	✓	✓	✗	✗
Abouzeid et al. [186]	Focused on hormonal, biological, and socio-environmental influences unique to women.	✓	✓	✓	✗	✓	✓	✗	✗
Zhao Y et al. [188]	Identified dietary patterns impacting women’s cardiovascular health, enriching preventive care strategies.	✓	✓	✗	✓	✓	✗	✗	✗
L. Cho et al. [189]	Showed how AI-based risk scores can identify disparities, improving gender-specific survival strategies.	✓	✓	✓	✗	✓	✓	✗	✗

By highlighting systemic improvements in diagnostic accuracy, Yan et al. [184] broaden this conversation and establish AI as a game-changing instrument in personalised medicine. This link illustrates a development in the use of AI, from the study of specific risk factors to the thorough integration of entire systems. Further, Zhao Y [188] et al. and Cho [189] et al. focus on particular risk factors, like dietary influences and gender-specific variations in heart attack fatalities. These studies benchmark against broader investigations like Morales Lara et al. [180] and Jonas et al. [181], which integrate lifestyle and gender-based considerations. This layer of comparison underscores how AI can bridge targeted risk assessments with broader health management strategies. Notably, while most studies explore the potential of AI, only one directly addresses the issue of bias in AI applications in healthcare, a critical area requiring further exploration. The presented collective benchmarking of these studies reveals the immense potential of AI in women’s CVD/Stroke research. By addressing nonlinear relationships among internal and external biomarkers (as shown in Figure 3), AI offers promising avenues for improved CVD/Stroke risk stratification and personalised care. Future efforts should focus on leveraging AI to balance holistic health insights with tailored interventions, addressing gender-specific gaps in CVD/Stroke research [190].

## 9. Recommendations to Improve CVD/Stroke Risk Stratification in Women

To improve AI models for CVD/stroke risk stratification in women patients, it is crucial to integrate diverse data types, including clinical, imaging, and genetic biomarkers, along with lifestyle information, to capture the multifactorial nature of risk [90]. Optimising hyperparameters and correcting data imbalances can improve forecast accuracy and fairness among risk categories [125]. Additionally, adding cost-effective surrogate indicators, such as carotid intima-media thickness (cIMT), and changing AI models for edge devices provides accessibility in resource-constrained environments and encourages broader and more equitable use. Each of these factors is covered in full below.

*i*. Comprehensive Data Integration

AI models must use a variety of data types, such as clinical factors, imaging results, genetic profiles, and biomarker information, to properly stratify the risk of CVD/stroke in women. Age, blood pressure, cholesterol, and hormone profiles are essential for risk assessment [33]. Imaging techniques for subclinical atherosclerosis and plaque features include carotid ultrasonography and echocardiography [40]. Biomarkers that reveal information about underlying physiological problems include lipid profiles, hormone levels, and inflammatory markers, such as C-reactive protein [39]. Furthermore, the construction of comprehensive models that consider the complex nature of CVD/stroke risks in women would be made possible by combining socio-environmental factors like healthcare access with patient lifestyle data like eating and activity patterns [41].

*ii*. Hyperparameter Optimisation

Hyperparameters play a major role in determining AI models’ performance and forecast accuracy [26]. Learning rates, activation functions, and dropout rates are among the parameters that can be systematically adjusted using methods like grid search, random search, and Bayesian optimisation [23]. Maintaining ideal hyperparameters contributes to improved risk classification accuracy, improved model generalisation, and decreased overfitting [191]. To better capture intricate, non-linear correlations between input features and CVD/stroke outcomes, optimisation procedures for DL models may also involve adjusting the architecture (such as the number of layers and neurons) [192].

*iii*. Balanced Risk Classes

Imbalanced datasets, where high-risk or low-risk groups are underrepresented, can create biased AI models, leading to poor generalisation and reduced accuracy in minority classes [193]. Techniques such as SMOTE, under sampling, and adaptive sampling strategies can create balanced datasets. This ensures that models have equal representation across control, low-risk, and high-risk groups, improving fairness and reliability [194]. Regular evaluation of the model’s performance metrics, such as the precision, recall, and F1 scores for each class, is essential to ensure that the classifier performs consistently across all risk categories [91].

*iv*. Edge Device Adaptability

To broaden the accessibility of AI-based solutions, models should be tuned for edge devices, particularly in underserved and resource-constrained locations [195]. Lightweight architectures, such as MobileNet or TensorFlow Lite, allow for deployment on mobile devices such as smartphones, tablets, and wearables [196,197]. Model pruning, quantisation, and knowledge distillation are techniques that can compress huge models while maintaining accuracy [198]. Real-time processing on edge devices makes risk assessment techniques available to doctors and patients in remote locations, improving the delivery of prompt and individualised care [37,199].

*v*. Surrogate Biomarkers for Cost-Effectiveness

Surrogate biomarkers, such as carotid intima-media thickness (cIMT), plaque area/burden, and maximum plaque height, offer a lower-cost alternative to more expensive diagnostic techniques [83]. These biomarkers are readily collected using non-invasive imaging techniques, such as carotid ultrasound, making them suitable for widespread application. Surrogate indicators have a strong predictive value, especially when paired with AI algorithms for detecting early CVD/stroke risk [31]. The use of affordable and accessible biomarkers assures that AI-driven risk categorisation is practical in healthcare settings, including low-resource contexts [200].

*vi*. Limitations of Current AI-Based Approaches

Despite the promise of AI-based risk stratification in women’s CVD, several methodological limitations constrain the clinical readiness of existing approaches and must be addressed in future research: (a) Overfitting in small or sex-imbalanced datasets: Many published AI models for women’s CVD risk were trained on datasets with limited sample sizes or with women constituting a minority subgroup [201]. In these settings, models are prone to overfitting, yielding inflated performance metrics that may not generalise to new populations or cohorts [202]. Regularisation, cross-validation on held-out sex-stratified sets, and reporting of confidence intervals for key metrics are essential [203]. (b) Limited external validation: Most described AI models have been validated on internal test sets derived from the same institution or study cohort as the training data [204]. External validation in geographically, ethnically, and clinically distinct women-only cohorts has rarely been reported and is a prerequisite for establishing generalizability [202]. (c) Calibration and transportability: Predictive models that are well-discriminating (high AUC) may still be poorly calibrated, systematically overestimating or underestimating risk. Calibration metrics (e.g., Brier score, calibration curves) [205] should be reported alongside discrimination measures, particularly when models are applied to populations outside the training distribution. (d) Algorithmic bias from population composition: Training datasets that underrepresent women or that reflect historical healthcare patterns in which women were undertreated or underdiagnosed can embed systematic biases into AI models [206]. Female-specific risk factors (e.g., APOs, autoimmune diseases, hormonal trajectories) are often absent from legacy cardiovascular datasets, further amplifying this bias [207,208,209]. (e) Data provenance: The source, completeness, and temporal span of training data critically influence model performance [210]. Models trained on electronic health records from high-income settings may not perform equitably in populations with different risk profiles, comorbidity patterns, or healthcare access, a particular concern for women in low-resource environments [211].

### Strengths, Weaknesses, and Future Directions

This narrative review integrates multiple risk domains—hormonal, immunological, clinical, and computational—providing a comprehensive and interdisciplinary perspective on women’s CVD/stroke risk. The inclusion of explainable AI frameworks, AI model architecture comparisons, and surrogate biomarker considerations reflects a clinically actionable synthesis that extends beyond descriptive summaries.

Several limitations of this review must be acknowledged. First, as a narrative rather than systematic review, the literature selection process may introduce selection or confirmation bias; studies reporting positive AI performance may be over-represented relative to null or negative findings. Second, the AI landscape evolves rapidly, and methodologies described in the reviewed studies may have been superseded or refined since the literature search was conducted. Third, the majority of reviewed studies were conducted in high-income settings with predominantly European, North American, or East Asian cohorts; generalizability to women from sub-Saharan Africa, South Asia, or Latin America, where CVD risk profiles and healthcare access differ substantially, is uncertain. Fourth, heterogeneity in outcome definitions, follow-up periods, and biomarker measurement methods across included studies limits cross-study comparisons. Fifth, publication bias toward significant results may cause the review to overstate the current capabilities of AI in women’s CVD risk prediction.

Future research should prioritise: (i) large-scale, prospective, externally validated AI studies in women-only or sex-stratified cohorts; (ii) longitudinal biomarker profiling incorporating hormonal trajectories, reproductive history, and immune biomarker dynamics; (iii) multi-ethnic and multi-continental validation studies to ensure equitable performance; (iv) integration of novel imaging modalities such as shear wave elastography and photoacoustic imaging; and (v) regulatory science investigations into the clinical deployment of AI-based CVD risk tools.

## 10. Conclusions

CVD and stroke continue to be the leading causes of death and long-term disability for women, yet our prevention strategies often fail to account for the female-specific attributes of these diseases. The presented study evaluates the biological link between women’s hormonal influences, autoimmune diseases and chronic inflammation, physiological and anatomical differences, pregnancy as a window to cardiovascular health, and CVD/stroke risk. Conventional risk models frequently miss the mark because they do not fully integrate factors like hormonal shifts, pregnancy complications, or unique socio-environmental stressors. This review examines the methodological potential of AI-driven models to address these gaps; however, it is essential to emphasise that the evidence base for AI-based CVD risk stratification in women-specific populations remains at an early stage. Most described models have been validated internally or in mixed-sex cohorts, and their performance in prospective, women-only, externally validated settings has not been established.

By incorporating gender-specific biomarkers into advanced machine learning frameworks, we can identify complex, nonlinear patterns that traditional statistics might overlook. What makes these tools particularly viable are practical innovations like “hyperparameter optimisation” and “balanced risk class handling”, a method that ensures the AI does not ignore rarer, high-risk cases in favour of the majority. Furthermore, by designing these models for edge devices and utilising affordable surrogate biomarkers, we can make high-level diagnostics both scalable and accessible.

While the integration of sex-specific biomarkers, advanced imaging, and AI-based frameworks represents an important direction for precision cardiovascular medicine, the current evidence base does not yet support clinical deployment. Achieving translational progress will require multi-institutional, prospective validation studies in diverse and sex-stratified female cohorts; transparent reporting of calibration and discrimination metrics; regulatory evaluation of clinical decision support tools; and continued interdisciplinary collaboration between data scientists, cardiologists, and women’s health specialists. The field is currently in an important exploratory phase; translating these methodological advances into validated, equitable, and clinically actionable tools will require sustained effort and rigorous scientific scrutiny.

## Figures and Tables

**Table 1:** Various Biomarkers and Disease Relevance [63].

Biomarker Category	Biomarkers	Disease Relevance
Metabolic Markers	Glucose, Fasting Glucose, Fasting Insulin, Haemoglobin A1c, Lipid Profile	Diabetes mellitus, CVD/Stroke
Hormonal Markers	Estradiol, Progesterone, FSH, LH, AMH	CVD/Stroke, Gynaecological Cancers, Reproductive Disorders
Markers of Inflammation	hs-CRP, IL-6, TNF-α	CVD/Stroke, Diabetes mellitus, Gynaecological Cancers
Autoimmune Markers	ANA, anti-dsDNA, Rheumatoid Factor (RF)	CVD/Stroke (autoimmune-related), Diabetes (T1DM)
Cancer Markers	CA-125, HE4, Alpha-fetoprotein (AFP), Therapy-associated Toxicity Markers	Gynaecological Cancers
Coagulation Markers	Fibrinogen, D-dimer	CVD/Stroke, Cancer-associated Thrombosis

CVD: Cardiovascular Disease, FSH: Follicle Stimulating Hormone, LH: Luteinizing Hormone, AMH: Anti-Müllerian Hormone, hs-CRP: High-sensitivity C-Reactive Protein, IL-6: Interleukin-6, TNF-α: Tumour Necrosis Factor-alpha, ANA: Antinuclear Antibody, anti-dsDNA: Anti-double-stranded DNA, RF: Rheumatoid Factor, T1DM: Type 1 Diabetes Mellitus, CA-125: Cancer Antigen 125, HE4: Human Epididymis Protein 4, AFP: Alpha-fetoprotein.

**Table 2:** Biomarkers and common clinical relevance [63].

Biomarker	Common Clinical Significance
CRP/hs-CRP	Systemic inflammation marker; elevated in CVD, T2DM, and indirectly in cancers
Lipid Profile (LDL, HDL, TC, TG)	Dyslipidaemia is a shared risk factor for CVD and T2DM; abnormal lipids may be associated with cancer risk.
LDH (Lactate Dehydrogenase)	Elevated LDH indicates tissue damage; it indirectly rises in CVD, T2DM (ischemia, metabolic stress)
IL-6	Pro-inflammatory cytokines are elevated in both vascular and metabolic diseases
D-dimer	Thrombotic risk marker, linked to CVD, vascular complications in T2DM
Total Cholesterol	Cardiometabolic risk marker

CRP/hs-CRP: C-Reactive Protein/High-sensitivity C-Reactive Protein, LDL: Low-Density Lipoprotein, HDL: High-Density Lipoprotein, TC: Total Cholesterol, TG: Triglycerides, LDH: Lactate Dehydrogenase, IL-6: Interleukin-6, D-dimer: Fibrin Degradation Product, Total Cholesterol: Total Cholesterol (sum of HDL, LDL, and VLDL).

**Figure A1:**
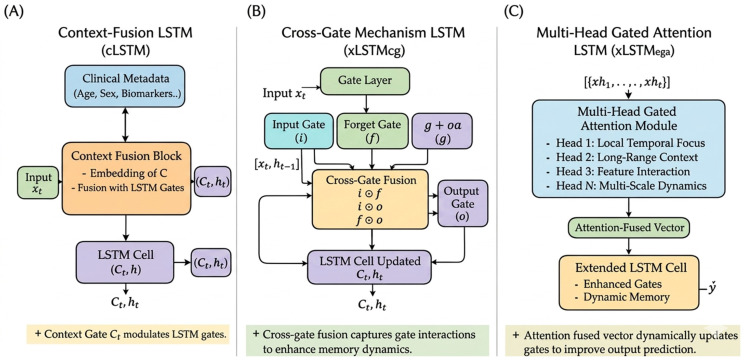
(**A**) Context-Fusion LSTM (cLSTM). (**B**) Cross-Gate Mechanism LSTM (xLSTMcg). (**C**) Multi-Head Gated Attention LSTM (xLSTMega).

## Data Availability

No new data were created or analyzed in this study. Data sharing is not applicable to this article.

## Abbreviations

ADASYN	Adaptive Synthetic Sampling	IL-6	Interleukin-6
AFP	Alpha-fetoprotein	LBBM	Laboratory-Based Biomarker Model
AI	Artificial Intelligence	LDH	Lactate Dehydrogenase
AMH	Anti-Müllerian Hormone	LDL	Low-Density Lipoprotein
ANA	Antinuclear Antibody	LH	Luteinizing Hormone
anti-dsDNA	Anti-double-stranded DNA	LIME	Local Interpretable Model-Agnostic Explanations
APOs	Adverse Pregnancy Outcomes	LSTM	Long Short-Term Memory
ASCVD	Atherosclerotic Cardiovascular Disease	MedUSE	Medication Use
AUC	Area Under Curve	ML	Machine Learning
CA-125	Cancer Antigen 125	OBBM	Office-Based Biomarker Model
cIMT	Carotid Intima-Media Thickness	OSA	Obstructive Sleep Apnea
CNN	Convolutional Neural Network	PA	Plaque Area/Plaque Burden
CVD	Cardiovascular Disease	PCA	Principal Component Analysis
D-dimer	Fibrin Degradation Product	PRISMA	Preferred Reporting Items for Systematic Reviews and Meta-Analyses
DE	Differential Evolution	PSO	Particle Swarm Optimisation
DL	Deep Learning	RA	Rheumatoid Arthritis
ECG	Electrocardiogram	RBBM	Radiomics-Based Biomarker Model
FCN	Fully Convolutional Network	ReLU	Rectified Linear Unit
FRS	Framingham Risk Score	RF	Random Forest
FSH	Follicle Stimulating Hormone	RNN	Recurrent Neural Network
GA	Genetic Algorithm	SHAP	Shapley Additive Explanations
GAN	Generative Adversarial Network	SLE	Systemic Lupus Erythematosus
GBBM	Genomics-Based Biomarker	SMOTE	Synthetic Minority Over-sampling Technique
GradCAM	Gradient-weighted Class Activation Mapping	T1DM	Type 1 Diabetes Mellitus
GRU	Gated Recurrent Unit	TC	Total Cholesterol
HDL	High-Density Lipoprotein	TG	Triglycerides
HE4	Human Epididymis Protein 4	TNF-α	Tumour Necrosis Factor-alpha
hs-CRP	High-sensitivity C-Reactive Protein		

## Appendix B. (Mathematical Equations of the LSTM Family)

### Appendix B.1. Context-Fusion LSTM (cLSTM)

Context-Fusion LSTM (cLSTM) extends the standard LSTM by explicitly incorporating contextual information (e.g., demographic, clinical, or imaging embeddings) into the temporal modelling process.

**Input Definitions**

- $x_{t}\in R^{d_{x}}$: primary sequential input at time step *t*
- $c_{t}\in R^{d_{c}}$: contextual feature vector at time step *t*
- $h_{t-1}$: hidden state from the previous time step
- $s_{t-1}$: cell state from the previous time step

**Context Fusion Equation**

(A1)
$${\tilde{x}}_{t}=[x_{t};c_{t}]$$

Equation (A1) concatenates the raw sequential input with contextual information, forming a context-aware input vector. By fusing context at this stage, all subsequent gates operate on information that reflects both current observations and prior/background knowledge.

**Forget Gate**

(A2)
$$f_{t}=\sigma (W_{f}{\tilde{x}}_{t}+U_{f}h_{t-1}+b_{f})$$

The forget gate determines how much of the previous memory $s_{t-1}$ should be retained.

- If $f_{t}\approx 1$, mentioned in Equation (A2), past information is preserved.
- If $f_{t}\approx 0$, mentioned in Equation (A2), outdated or irrelevant memory is discarded.
- Contextual features influence this decision directly.

**Input Gate**

(A3)
$$i_{t}=\sigma (W_{i}{\tilde{x}}_{t}+U_{i}h_{t-1}+b_{i})$$

The input gate shown in Equation (A3) controls how much new information should be written into memory. It ensures that only contextually relevant features are incorporated into the cell state.

**Output Gate**

(A4)
$$o_{t}=\sigma (W_{o}{\tilde{x}}_{t}+U_{o}h_{t-1}+b_{o})$$

The output gate shown in Equation (A4) regulates how much of the internal cell state contributes to the hidden state $h_{t}$, which is passed forward in the sequence.

**Candidate Cell State**

(A5)
$${\tilde{s}}_{t}=tanh(W_{s}{\tilde{x}}_{t}+U_{s}h_{t-1}+b_{s})$$

This Equation (A5) computes a candidate memory update that incorporates new information derived from the current context-aware input and the past hidden state.

**Cell State Update**

(A6)
$$s_{t}=f_{t}⊙s_{t-1}+i_{t}⊙{\tilde{s}}_{t}$$

The updated cell state (Equation (A6)) is a weighted combination of retained past memory ($f_{t}⊙s_{t-1}$) and newly acquired information ($i_{t}⊙{\tilde{s}}_{t}$).

**Hidden State Update**

(A7)
$$h_{t}=o_{t}⊙tanh(s_{t})$$

The hidden state (Equation (A7)) represents the observable output of the LSTM at time t and summarises the context-aware temporal information.

### Appendix B.2. Cross-Gate Mechanism LSTM (xLSTM_cg_)

xLSTM*_cg_* is designed for multimodal learning, where different modalities dynamically regulate each other’s influence.

**Multimodal Inputs**

- $x_{t}^{A}$: modality A (e.g., vital signs)
- $x_{t}^{B}$: modality B (e.g., laboratory biomarkers)

**Cross-Gate from *B* to *A***

(A8)
$$g_{t}^{A\leftarrow B}=\sigma (W_{AB}x_{t}^{B}+b_{AB})$$

This gate (Equation (A8)) allows modality *B* to determine how much information from modality *A* should be retained.

**Cross-Gate from *A* to *B***

(A9)
$$g_{t}^{B\leftarrow A}=\sigma (W_{BA}x_{t}^{A}+b_{BA})$$

This gate (Equation (A9)) performs the reciprocal operation, enabling modality *A* to regulate modality *B*.

**Cross-Modulated Inputs**

(A10)
$${\hat{x}}_{t}^{A}=g_{t}^{A\leftarrow B}⊙x_{t}^{A}$$

(A11)
$${\hat{x}}_{t}^{B}=g_{t}^{B\leftarrow A}⊙x_{t}^{B}$$

Each modality (Equations (A10) and (A11)) is selectively filtered using information from the other modality, suppressing noise and emphasising complementary patterns.

**Fusion of Modalities**

(A12)
$${\tilde{x}}_{t}=[{\hat{x}}_{t}^{A};{\hat{x}}_{t}^{B}]$$

The gated modalities are concatenated (Equation (A12)) to form a unified representation that reflects balanced multimodal information.

**LSTM Gates and Updates**

(A13)
$$f_{t}=\sigma (W_{f}{\tilde{x}}_{t}+U_{f}h_{t-1}+bf)\;i_{t}=\sigma (W_{i}{\tilde{x}}_{t}+U_{i}h_{t-1}+bi)\;o_{t}=\sigma (W_{o}{\tilde{x}}_{t}+U_{o}h_{t-1}+bo)\;{\tilde{s}}_{t}=tanh(W_{s}{\tilde{x}}_{t}+U_{s}h_{t-1}+bs)\;s_{t}=f_{t}⊙s_{t-1}+i_{t}⊙{\tilde{s}}_{t}\;h_{t}=o_{t}⊙tanh(s_{t})$$

Once multimodal fusion is complete, the network follows standard LSTM dynamics shown in Equation (A13) to model temporal dependencies.

### Appendix B.3. Multi-Head Gated Attention LSTM (xLSTMega)

xLSTM*ega* enhances LSTM by incorporating long-range temporal dependencies via gated multi-head attention.

**Hidden States**

(A14)
$$H_{t-1}=[h_{1},\ldots ,h_{t-1}]$$

The matrix represented in (Equation (A14)) stores all previous hidden states, serving as the memory pool for attention.

**Query, Key, and Value Projections**

(A15)
$$Q_{t}^{\left(k\right)}=W_{Q}^{\left(k\right)}h_{t-1},K^{\left(k\right)}=W_{K}^{\left(k\right)}H_{t-1},V^{\left(k\right)}=W_{V}^{\left(k\right)}H_{t-1}$$

Each attention head *k* projects the hidden states into subspaces to capture different temporal patterns.

**Attention Weights**

(A16)
$$\alpha_{t}^{\left(k\right)}=\mathrm{softmax}\left(\frac{Q_{t}^{\left(k\right)}K^{(k)T}}{\sqrt{d_{k}}}\right)$$

(Equation (A16)) assigns importance scores to past time steps, emphasising the most relevant historical information.

**Attention Head Output**

(A17)
$$z_{t}^{\left(k\right)}=\alpha_{t}^{\left(k\right)}V^{\left(k\right)}$$

Each attention head produces shown in (Equation (A17)) a context vector summarising relevant historical information.

**Multi-Head Fusion**

(A18)
$$z_{t}=\mathrm{Concat}(z_{t}^{\left(1\right)},\ldots ,z_{t}^{\left(K\right)})$$

Outputs from all heads shown in (Equation (A18)) are concatenated to form a comprehensive temporal context representation.

**Gated Attention Integration**

(A19)
$$g_{t}=\sigma (W_{g}[x_{t};z_{t}])$$

(A20)
$${\tilde{x}}_{t}=g_{t}⊙z_{t}+(1-g_{t})⊙x_{t}$$

The gating mechanism shown in (Equation (A19)) adaptively balances current input signals and attention-derived historical context, preventing over-reliance on either source.

**LSTM Update**

(A21)
$$f_{t}=\sigma \left(W_{f}{\tilde{x}}_{t}+U_{f}h_{t-1}\right)\;i_{t}=\sigma (W_{i}{\tilde{x}}_{t}+U_{i}h_{t-1})\;o_{t}=\sigma (W_{o}{\tilde{x}}_{t}+U_{o}h_{t-1})\;{\tilde{s}}_{t}=tanh(W_{s}{\tilde{x}}_{t}+U_{s}h_{t-1})\;s_{t}=f_{t}⊙s_{t-1}+i_{t}⊙{\tilde{s}}_{t}\;h_{t}=o_{t}⊙tanh(s_{t})$$

The gated attention output is treated as the effective input to the LSTM, Equation (A16), ensuring stable long-term memory learning.

In summary, the cLSTM integrates contextual knowledge at the input level, xLSTM*cg* enables adaptive cross-modal interaction, and xLSTM*ega* augments LSTM with gated multi-head temporal attention, collectively enabling robust and interpretable modelling of complex multimodal time-series data.

## Appendix C

**Table A1:** Comparison of the LSTM model family.

Model	Key Mechanism	Strength of Temporal Modelling	Noise Robustness	Context Integration Capability	CC	Representative Applications	Relevant References
cLSTM	Context fusion via auxiliary covariate embedding integrated into LSTM gates	Moderate to High	Moderate	High (explicit context-aware gates)	Low	Patient-specific modelling, metadata-driven risk prediction	Hochreiter et al. [212] and Lipton et al. [213]
xLSTMcg	Cross-gate mechanism with structured interactions between input, forget, and output gates	High	High (selective gating suppresses noise)	Moderate	Medium	Biomedical signal processing (ECG, EEG, PPG), long-range temporal dependency tasks	Greff et al. [214], Chung et al. [215]
xLSTMega	Multi-head gated attention + dynamic memory controller + parallel temporal pathways	Very High	Very High	Very High	High	Multi-modal clinical data, EHR longitudinal modelling, and multi-omics sequence integration	Vaswani et al. [216], Bahdanau et al. [217]

cLSTM: Context-aware Long Short-Term Memory, xLSTMcg: Extended Long Short-Term Memory with Context Gating, xLSTMega: Extended Long Short-Term Memory with Enhanced Gated Attention, CC: Computational Cost, ECG: Electrocardiogram, EEG: Electroencephalogram, PPG: Photoplethysmogram, EHR: Electronic Health Records.
